# Classic psychedelics, health behavior, and physical health

**DOI:** 10.1177/20451253221135363

**Published:** 2022-11-30

**Authors:** Otto Simonsson, Peter S. Hendricks, Richard Chambers, Walter Osika, Simon B. Goldberg

**Affiliations:** Center for Psychiatry Research, Department of Clinical Neuroscience, Karolinska Institute, Tomtebodavägen 18A, 171 77, Stockholm, Sweden; Department of Sociology, University of Oxford, Oxford, 42-43 Park End Street, OX1 1JD, UK; Department of Health Behavior, School of Public Health, University of Alabama at Birmingham, 1665 University Boulevard, 35233, Birmingham, AL, USA; Department of Health Behavior, School of Public Health, University of Alabama at Birmingham, Birmingham, AL, USA; Monash Centre for Consciousness & Contemplative Studies, Monash University, Melbourne, VIC, Australia; Center for Psychiatry Research, Department of Clinical Neuroscience, Karolinska Institute, Stockholm, Sweden; Department of Counseling Psychology, University of Wisconsin–Madison, Madison, WI, USA

**Keywords:** behavior, BMI, health, psilocybin, psychedelics

## Abstract

**Background:**

Preliminary evidence suggests that classic psychedelics may be effective in the treatment of some psychiatric disorders, yet little remains known about their effects on health behavior and physical health.

**Objectives:**

The purpose of this study was to investigate associations of lifetime classic psychedelic use and psychological insight during one’s most insightful classic psychedelic experience with health behavior and physical health.

**Methods:**

Using data representative of the US population with regard to sex, age, and ethnicity (*N* = 2822), this study examined associations of lifetime classic psychedelic use and psychological insight with health behavior and physical health.

**Results:**

Lifetime classic psychedelic use was associated with more healthy tobacco-related and diet-related behavior (*β* = 0.05 and 0.09, respectively). Among lifetime classic psychedelic users (*n* = 613), greater Psychological Insight Questionnaire (PIQ) total scale, PIQ Avoidance and Maladaptive Patterns (AMP) subscale, and PIQ Goals and Adaptive Patterns (GAP) subscale scores were each associated with higher odds of more healthy exercise-related behavior [adjusted odds ratios (aOR) (95% confidence interval, CI = 1.38 (1.13–1.68), 1.38 (1.13–1.68), and 1.32 (1.10–1.60), respectively] and higher odds of having a healthy body mass index (BMI) [aOR (95% CI) = 1.32 (1.07–1.63), 1.36 (1.10–1.69), and 1.23 (1.01–1.50), respectively], and greater GAP subscale scores were associated with more healthy diet-related behavior (*β* = 0.10). All PIQ scales were positively associated with some health behavior improvements (overall, diet, exercise) attributed to respondents’ most insightful classic psychedelic experience (*β* = 0.42, 0.18, and 0.17; *β* = 0.40, 0.19, and 0.17; and *β* = 0.40, 0.15, and 0.15, respectively), but only PIQ total scale and AMP subscale scores were positively associated with alcohol-related health behavior improvements (*β* = 0.13 and 0.16, respectively).

**Conclusion:**

Although these results cannot demonstrate causality, they suggest that psychological insight during a classic psychedelic experience may lead to positive health behavior change and better physical health in some domains, in particular in those related to weight management.

Preliminary evidence suggests that classic psychedelics (i.e. serotonin 2A receptor agonists such as mescaline and psilocybin) may be effective as treatments for some mental health conditions,^1^ but recent research has also found links between classic psychedelic use and better physical health. For example, population-based studies with representative samples of the US adult population have revealed associations between lifetime classic psychedelic use and lower odds of being overweight or obese as well as lower odds of having heart disease, diabetes, and hypertension in the past year.^2–4^

It is plausible that the associations between lifetime classic psychedelic use and better physical health can, at least in part, be explained by changes in health behavior.^5^ For instance, one qualitative analysis revealed that many participants in a clinical trial reported spontaneous health behavior changes following administration of psilocybin.^6^ Other studies with cross-sectional research designs indicate that lifetime classic psychedelic use may be associated with positive health behavior related to alcohol, tobacco, diet, and exercise,^7,8^ but such links have not yet been examined in nationally representative samples free of significant self-selection bias.

If classic psychedelic use can indeed facilitate health behavior change, it would be important to understand the underlying psychological mechanisms. While several measures of the acute classic psychedelic experience show promise as predictors of long-term outcomes (e.g. Mystical Experience Questionnaire,^9,10^ Ego Dissolution Inventory,^11,12^ and Challenging Experience Questionnaire^13,14^), recent research suggests that psychological insight may be a common feature of the classic psychedelic experience and particularly important for therapeutic efficacy.^7,15,16^

There are currently two self-report measures of psychological insight that have been used in research on classic psychedelics. The first, the Psychological Insight Questionnaire (PIQ), was designed to measure psychological insight during the acute classic psychedelic experience.^15^ The second, the Psychological Insight Scale, was designed to capture psychological insight that crystallizes after the acute classic psychedelic experience.^16^ The shared associations of these two self-report measures are not yet known, however, and neither has so far been used to investigate associations with health behavior.

Using a sample representative of the US adult population with regard to sex, age, and ethnicity from a large-scale online survey (*N* = 2822), the present study investigated the associations of lifetime classic psychedelic use and psychological insight during respondents’ most insightful classic psychedelic experience with health behavior (alcohol, tobacco, diet, exercise) and physical health [body mass index (BMI) and non-communicable disease (NCD) in the past year]. We hypothesized that lifetime classic psychedelic use and psychological insight during respondents’ most insightful experience using a classic psychedelic would be associated with more healthy tobacco-related, alcohol-related, diet-related, and exercise-related behaviors. We also hypothesized that lifetime classic psychedelic use and psychological insight during respondents’ most insightful experience using a classic psychedelic would be associated with higher odds of having a healthy BMI and lower odds of having had a NCD in the past year.

## Participants and procedure

The study (hypotheses, design plan, sampling plan, variables, and analysis plan) was preregistered on the Open Science Framework (OSF) at https://osf.io/ekyan and https://osf.io/5vtj2. The sample size was determined using linear multiple regression (fixed model, R2 increase) in GPower,^17^ which revealed that 395 lifetime classic psychedelic users would achieve 80% power to detect small effect sizes with an alpha of .05. Assuming similar prevalence of lifetime classic psychedelic use in the US adult population as recent investigations (~14%),^2^ we estimated that 2800 total participants would be necessary to get approximately 395 lifetime classic psychedelic users in the sample. We, therefore, aimed to recruit 2800 participants.

The participants were US residents who were 18 years or older and were recruited between 1 October 2021 and 9 October 2021 through Prolific Academic (https://app.prolific.co), which is a participant recruitment platform for researchers. The platform offers a representativeness function that uses proportionate stratification on three census-matched factors – sex (male, female), age (18–27, 28–37, 38–47, 48–57, and 58+), and ethnicity (White, Mixed, Asian, Black, Other) – to reflect the demographic distribution of the US adult population. If participants do not complete the study (*n* = 131 non-completers in this study), the platform’s algorithms dynamically adjust sampling requirements over time in order to deliver the most representative sample possible. The study description in recruitment materials focused on health behavior and did not mention classic psychedelic use (see Supplemental Materials for recruitment materials) to avoid potential self-selection bias. All participants gave informed consent digitally before being asked about demographic characteristics, substance use, health behavior, and health status. Participants who reported lifetime classic psychedelic use (*n* = 613) were also asked additional questions related to their use of classic psychedelics (e.g. psychological insight). Study completion resulted in US$2.20 payment. Study procedures were determined to be exempt from review by the Institutional Review Board at the University of Wisconsin–Madison (reference number: 2021-1128). All procedures performed involving human participants were in accordance with the ethical standards of the 1964 Helsinki declaration and its later amendments or comparable ethical standards.

### Measures

#### Demographics

All respondents were asked to report age in years, gender, ethnoracial identity, sexual orientation, educational attainment, annual household income, marital status, and engagement in risky behavior.

#### Substance use

All respondents were also asked to report lifetime use of cocaine, sedatives, pain relievers, marijuana, phencyclidine (PCP), 3,4-methylenedioxymethamphetamine (MDMA/ecstasy), inhalants, smokeless tobacco, pipe tobacco, cigar, and cigarettes daily, and age of first alcohol use. (Due to a technical error, data were not collected on lifetime use of other stimulants, heroin, and tranquilizers.)

#### Tobacco-related health behavior

To assess tobacco-related health behavior, respondents were asked to complete the 7-item Fagerström Test for Cigarette Dependence,^18^ a widely used and valid measure of the severity of nicotine dependence related to cigarette smoking. The items (e.g. ‘How soon after you wake up do you smoke your first cigarette?’) had either two response options (scored 0–1) or four response options (scored 0–3), with 10 as the maximum score. Respondents were coded as 0 if they reported no current use of cigarettes. The scores were reverse-scored, and greater scores, therefore, reflect less cigarette dependence (i.e. more healthy tobacco-related behavior).

#### Alcohol-related health behavior

To assess alcohol-related health behavior, respondents were asked to complete the 3-item Alcohol Use Disorders Identification Test–Concise (AUDIT-C),^19^ which measures alcohol-related risk behavior. The items (e.g. ‘How often do you have a drink containing alcohol?’) had five response options (scored 0–4), with 12 as the maximum total score. Respondents were coded as 0 if they reported no current use of alcohol. The scores were reverse-scored, and greater scores, therefore, reflect less problematic drinking behavior (i.e. more healthy alcohol-related behavior).

#### Exercise-related behavior

To assess exercise-related behavior, respondents were asked to complete the 7-item International Physical Activity Questionnaire–Short Form (IPAQ-SF),^20^ which measures physical activity in adults. The items (e.g. ‘During the last 7 days, on how many days did you do vigorous physical activities such as heavy lifting, digging, aerobics, or fast bicycling?’) assessed the number of days and the number of minutes each day that respondents engaged in vigorous activity, moderate-intensity activity, and walking. The responses were used to categorize behavior into three physical activity categories: low, medium, and high. Greater scores, therefore, reflect more healthy exercise-related behavior.

#### Diet-related behavior

To assess diet-related behavior, respondents were asked to complete the 8-item Starting the Conversation scale (STC),^21^ which measures dietary quality. The items (e.g. ‘Over the past few months, how many times a week did you eat fast food meals or snacks?’) had three response options (scored 0–2), with 16 as the maximum total score. The scores were reverse-scored, and greater scores, therefore, reflect more healthy diet-related behavior.

#### BMI

To assess BMI, respondents were asked to report height and weight, which was used to calculate BMI. As indicated in the preregistration, respondents who had a BMI between 18.5 and 25 were coded as positive for healthy BMI, whereas those who had a BMI outside of this range were coded as negative (0 = unhealthy BMI and 1 = healthy BMI). This evaluation of BMI contrasts with the coding of BMI in previous research (underweight, normal, overweight, obese – Class 1, obese – Class 2, and obese – Class 3),^2^ but a dichotomous variable was chosen in light of the much smaller sample size in this study.

#### NCD in the past year

Consistent with previous research,^3^ to assess NCD in the past year, respondents were asked whether they were told by a doctor or other medical professional that they had cancer, heart disease, high blood pressure, or type 2 diabetes in the past 12 months. As indicated in the preregistration, respondents who reported any of these NCDs were coded as positive for NCD in the past year, whereas those indicating that they had not had any of those NCDs in the past year were coded as negative (0 = no NCD in the past year and 1 = NCD in the past year). Previous research has measured each NCD separately (e.g. hypertension, heart disease, and diabetes),^3,4^ but collapsing all NCDs into one variable was chosen in light of the much smaller sample size in this study.

#### Lifetime classic psychedelic use

Consistent with previous research,^3^ all respondents were asked to report which, if any, of the following classic psychedelics they had ever used: ayahuasca, *N*, *N*-dimethyltryptamine (DMT), lysergic acid diethylamide (LSD), mescaline, peyote, San Pedro, and psilocybin. Respondents who reported having used any of these were coded as positive for lifetime classic psychedelic use, whereas those indicating that they had never used any of these substances were coded as negative (0 = No and 1 = Yes).

#### Psychological insight

Respondents who reported lifetime classic psychedelic use were asked to look back on their most insightful experience using a classic psychedelic and complete the 23-item PIQ,^15^ which was designed to capture the subjective experience of insight during the acute effects of a classic psychedelic (e.g. ‘Discovered new actions that may help me achieve my goals’ and ‘Discovered how aspects of my life are affecting my well-being’). The responses were rated from 0 (No; not at all) to 5 (Extremely) on a Likert-type scale. The mean of all items comprises the overall PIQ total score scale (coefficient alpha = 0.98 in the current sample). There are also two subscales: Avoidance and Maladaptive Patterns (AMP; coefficient alpha = 0.97 in the current sample, e.g. ‘Awareness of uncomfortable or painful feelings I previously avoided’) and Goals and Adaptive Patterns (GAP; coefficient alpha = 0.95 in the current sample, e.g. ‘Realized the importance of my life’). Author-constructed items also asked respondents whether they thought their most insightful experience using a classic psychedelic and their contemplation of that experience had led to changes in their health behavior (overall, tobacco, alcohol, diet, exercise), with responses rated on a 0-point (worsened a lot) to 100-point (improved a lot) slider scale.

#### Statistical analyses

We used linear (for alcohol-, tobacco-, and diet-related behavior), ordered logistic (for exercise-related behavior), and logistic (for BMI and NCD in the past year) regression models to evaluate associations between classic psychedelic-related variables and variables related to health behaviors. All models included control variables related to demographics and substance use: age in years (18–25, 26–34, 35–49, 50–64, or 65 or older), gender (male, female, transgender/non-binary), ethnoracial identity (non-Hispanic White, non-Hispanic African American, non-Hispanic Native American/Alaska Native, non-Hispanic Native Hawaiian/Pacific Islander, non-Hispanic Asian, non-Hispanic more than one race, Hispanic), sexual orientation (heterosexual, bisexual, gay/lesbian, other), educational attainment (some high school or less, high school graduate or equivalent, some college/community college degree, Bachelor’s degree or higher), annual household income (less than US$20,000, US$20,000–49,999, US$50,000–74,999, US$75,000, or more), marital status (married/living with a partner/in a long-term relationship, widowed, divorced/separated, not married/single), lifetime engagement in risky behavior (never, seldom, sometimes, always), lifetime use of cocaine (yes, no), sedatives (yes, no), pain relievers (yes, no), marijuana (yes, no), PCP (yes, no), 3,4-MDMA/ecstasy (yes, no), inhalants (yes, no), smokeless tobacco (yes, no), pipe tobacco (yes, no), cigars (yes, no), and cigarettes daily (yes, no), and age of first alcohol use (less than 13 years, 13–19 years, more than 20 years, never used alcohol). Overall PIQ and the two (GAP/AMP) subscales were z-scored to standardize scores and aid comparison.

## Results

Supplemental Table 1 shows descriptive statistics of lifetime classic psychedelic users *versus* non-lifetime classic psychedelic users. As seen in the table, lifetime classic psychedelic use was, for example, more common among men, individuals with greater self-reported engagement in risky behavior, and individuals who reported lifetime use of other illicit substances. Table 1 presents results from the regression models testing the unique associations between lifetime classic psychedelic use, psychological insight, and health behavior. As demonstrated in the table, lifetime classic psychedelic use was very modestly associated with more healthy tobacco-related and diet-related behavior, but no association was observed with alcohol-related and exercise-related behavior. Among the respondents who reported lifetime classic psychedelic use (*n* = 613), greater PIQ total scale scores, AMP subscale scores, and GAP subscale scores were each modestly associated with higher odds of more healthy exercise-related behavior. Greater GAP subscale scores were also modestly associated with more healthy diet-related behavior, but no other associations were observed (see Supplemental Table 2 for exploratory analyses).

**Table 1.** Classic psychedelic use, psychological insight, and health behavior.

	Tobacco		Alcohol		Diet		Exercise		*N*
	*β*	*p*	*β*	*p*	*β*	*p*	aOR (95% CI)	*p*	
Lifetime use	0.05	**0.034**	0.03	0.157	0.09	**<0.001**	1.02 (0.80–1.29)	0.895	2822
PIQ scale	0.03	0.557	0.02	0.717	0.06	0.177	1.38 (1.13–1.68)	**0.001**	613
AMP subscale	0.01	0.825	0.02	0.673	0.03	0.547	1.38 (1.13–1.68)	**0.002**	613
GAP subscale	0.04	0.293	0.01	0.811	0.10	**0.022**	1.32 (1.10–1.60)	**0.003**	613

AMP, Avoidance and Maladaptive Patterns; aOR, adjusted odds ratios; BMI, body mass index; GAP, Goals and Adaptive Patterns; PIQ, Psychological Insight Questionnaire; β, standardized coefficients. Significant results (*p* < 0.05) highlighted in bold.

β and aORs are adjusted for age in years, gender, ethnoracial identity, sexual orientation, educational attainment, annual household income, marital status, self-reported engagement in risky behavior, lifetime use of cocaine, sedatives, pain relievers, marijuana, phencyclidine (PCP), 3,4-methylenedioxymethamphetamine (MDMA/ecstasy), inhalants, smokeless tobacco, pipe tobacco, cigars, and cigarettes daily, and age of first alcohol use.

Table 2 displays results from the regression models testing the unique associations between health behavior change attributed to respondents’ most insightful classic psychedelic experience and degree of psychological insight during that experience. As indicated in the table, greater PIQ total scale scores, AMP subscale scores, and GAP subscale scores were each moderately to strongly associated with overall health behavior improvements, and modestly associated with improvements in diet and exercise attributed to the most insightful classic psychedelic experience. Greater PIQ total scale scores and AMP subscale scores were also modestly associated with alcohol-related health behavior improvements attributed to the most insightful classic psychedelic experience. No association was observed with health behavior improvements related to tobacco in any of the models.

**Table 2.** Health behavior change attributed to most insightful psychedelic experience.

	Overall		Tobacco		Alcohol		Diet		Exercise		*N*
	*β*	*p*	*β*	*p*	*β*	*p*	*β*	*p*	*β*	*p*	
PIQ scale	0.42	**<0.001**	0.00	0.992	0.13	**0.005**	0.18	**<0.001**	0.17	**<0.001**	613
AMP subscale	0.40	**<0.001**	0.02	0.640	0.16	**0.001**	0.19	**<0.001**	0.17	**<0.001**	613
GAP subscale	0.40	**<0.001**	–0.03	0.490	0.09	0.055	0.15	**0.001**	0.15	**0.001**	613

AMP, Avoidance and Maladaptive Patterns; GAP, Goals and Adaptive Patterns; PIQ, Psychological Insight Questionnaire; β, standardized coefficients. Significant results (*p* < 0.05) highlighted in bold.

β are adjusted for age in years, gender, ethnoracial identity, sexual orientation, educational attainment, annual household income, marital status, self-reported engagement in risky behavior, lifetime use of cocaine, sedatives, pain relievers, marijuana, phencyclidine (PCP), 3,4-methylenedioxymethamphetamine (MDMA/ecstasy), inhalants, smokeless tobacco, pipe tobacco, cigars, and cigarettes daily, and age of first alcohol use.

Table 3 shows results from the regression models testing the unique associations between lifetime classic psychedelic use, psychological insight, and physical health. As shown in the table, lifetime classic psychedelic use was not associated with BMI, but greater PIQ total scale scores, AMP subscale scores, and GAP subscale scores were associated with modestly higher odds of having a healthy BMI. No association was observed with NCD in the past year in any of the models (see Supplemental Table 3 for exploratory analyses).

**Table 3.** Classic psychedelic use, psychological insight, and physical health.

	Healthy BMI		NCD in the past year		*N*
	aOR (95% CI)	*p*	aOR (95% CI)	*p*	
Lifetime use	1.06 (0.82–1.37)	0.662	0.89 (0.65–1.23)	0.487	2822
PIQ scale	1.32 (1.07–1.63)	**0.009**	1.23 (0.95–1.61)	0.115	613
AMP subscale	1.36 (1.10–1.69)	**0.004**	1.22 (0.93–1.60)	0.144	613
GAP subscale	1.23 (1.01–1.50)	**0.043**	1.22 (0.95–1.55)	0.120	613

aOR: adjusted odds ratios; BMI, body mass index; GAP, Goals and Adaptive Patterns; PIQ, Psychological Insight Questionnaire. Significant results (*p* < 0.05) highlighted in bold.

aOR are adjusted for age in years, gender, ethnoracial identity, sexual orientation, educational attainment, annual household income, marital status, self-reported engagement in risky behavior, lifetime use of cocaine, sedatives, pain relievers, marijuana, phencyclidine (PCP), 3,4-methylenedioxymethamphetamine (MDMA/ecstasy), inhalants, smokeless tobacco, pipe tobacco, cigars, and cigarettes daily, and age of first alcohol use. Note: due to empty cells and collinearity in Stata, ethnoracial identity was dropped from regressions with lifetime use and NCD in the past year while educational attainment was dropped from regressions with psychological insight and NCD in the past year.

## Discussion

The present study investigated the associations between lifetime classic psychedelic use, psychological insight during respondents’ most insightful experience using a classic psychedelic, health behavior, and physical health in a representative sample of the US adult population. In covariate-adjusted regressions, results revealed very modest but statistically significant associations between lifetime classic psychedelic use and more healthy tobacco-related and diet-related behaviors. Among those who reported having used classic psychedelics at least once, greater PIQ total scale scores were modestly associated with higher odds of more healthy exercise-related behavior and higher odds of having a healthy BMI. Greater AMP and GAP subscale scores were also modestly associated with more healthy exercise-related behavior and higher odds of having a healthy BMI, although only greater GAP subscale scores were associated with more healthy diet-related behavior, with an effect modest in size. In regard to health behavior change attributed to respondents’ most insightful classic psychedelic experience, greater PIQ total scale scores, AMP subscale scores, and GAP subscale scores were moderately to strongly associated with greater overall health behavior improvements and greater diet-related and exercise-related health behavior improvements. Only greater PIQ total scale scores and AMP subscale scores were associated with greater alcohol-related health behavior improvements, with effects modest in size.

The findings in this study suggest that psychological insight induced by classic psychedelic use may contribute to positive health behavior change and better physical health in some domains. Notably, one of the most consistent findings was the links between psychological insight during respondents’ most insightful classic psychedelic experience using a classic psychedelic with more healthy diet-related and exercise-related health behavior and having a healthy BMI, which are variables particularly relevant to obesity and its adverse health effects. This suggests that classic psychedelics could be a promising research avenue in weight management, at least to the degree that classic psychedelics elicit insight. It is, therefore, important to investigate how existing treatments can be leveraged to maximize insight (e.g. using meditative practices designed to cultivate self-knowledge and insight as part of the treatment).^22^

While the relationships with alcohol-related and tobacco-related health behavior were less clear, previous research suggests that classic psychedelic use can lead to alcohol and tobacco smoking cessation.^7,23,24^ Such studies have recruited participants with a history of problematic alcohol or tobacco use, which contrasts with the more representative sample used in this study. The associations (and the lack thereof) in this study with alcohol-related and tobacco-related health behavior should, therefore, be interpreted with caution, with floor effects potentially impacting results.

The null findings on lifetime classic psychedelic use and physical health (healthy BMI, NCD in the past year) contrast with previous results from large, nationally representative studies in the United States.^2,3,4^ It is important to note that the previous studies had approximately 60–130 times more respondents and, therefore, had more statistical power to detect significant relationships, but there were also important differences in how physical health was measured and coded (e.g. BMI was coded as a dichotomous variable in this study whereas BMI was coded as a six-level variable in previous research),^2^ which further limits the comparisons that can be made between studies.

There are several further limitations in the research design that should be considered when interpreting the results. First, the sample was stratified across sex, age, and ethnicity to reflect the US adult population, but it does not appear to have been representative on other variables related to health behavior and physical health (most notably the socioeconomic indicators educational attainment and annual household income). The percentage of participants who reported lifetime classic psychedelic use (22%) was similar to the prevalence of lifetime classic psychedelic use (23%) in another study using Prolific Academic for study recruitment,^25^ but it was still higher than the prevalence in the United States (14%) in previous research using the National Survey on Drug Use and Health (NSDUH).^2^ This may be because the current sample was of higher socioeconomic status than the NSDUH sample^26^ and also because the previous research using the NSDUH likely underestimates ayahuasca and DMT use.^25^ Second, the questionnaire did not include items related to set and setting of the most insightful classic psychedelic experience, which could have been used to identify context-dependent variables related to psychological insight, health behavior, and physical health. Since previous research has found set and setting to be an important aspect of the classic psychedelic experience,^27,28^ it would, therefore, be interesting to explore the contribution of these variables in future research. Third, given the cross-sectional study design, the results cannot be used to make conclusive causal inferences. It is possible that lifetime classic psychedelic use or greater psychological insight leads to positive health behavior change, but it may be equally possible that healthier people are more likely to have used classic psychedelics over their lifetime and to have had greater psychological insight during such experiences. Finally, the covariate-adjusted regressions controlled for several potential confounders, but it is possible that other uncontrolled variables (e.g. personality traits) may have affected the associations reported here. Future research should use longitudinal designs and attempt to elucidate the potential causal links between classic psychedelic use, psychological insight, health behavior, and physical health.

## Conclusion

Preliminary evidence suggests that classic psychedelics may be effective in the treatment of some mental health conditions, yet little remains known about the effects of classic psychedelics on health behavior and physical health. The findings in the present study suggest that the subjective experience of psychological insight elicited by classic psychedelics may contribute to positive health behavior change and better physical health in some domains, in particular in those related to weight management. This study serves as a springboard for future longitudinal designs, including randomized controlled trials, that can better interrogate potential causal pathways of classic psychedelics on health behavior change and physical health.

## Supplemental Material

sj-docx-1-tpp-10.1177_20451253221135363 – Supplemental material for Classic psychedelics, health behavior, and physical health Click here for additional data file. Supplemental material, sj-docx-1-tpp-10.1177_20451253221135363 for Classic psychedelics, health behavior, and physical health by Otto Simonsson, Peter S. Hendricks, Richard Chambers, Walter Osika and Simon B. Goldberg in Therapeutic Advances in Psychopharmacology

## Declarations

**Ethics approval and consent to participate:** Study procedures were determined to be exempt from review by the Institutional Review Board at the University of Wisconsin–Madison (reference number: 2021-1128). All procedures performed involving human participants were in accordance with the ethical standards of the 1964 Helsinki declaration and its later amendments or comparable ethical standards. Informed consent was obtained digitally from all individual participants included in the study.

**Consent for publication:** Not applicable.

**Availability of data and materials:** The data and Stata syntax are available at the Open Science Framework: https://doi.org/10.6084/m9.figshare.21490059.v1 [Data] and https://doi.org/10.6084/m9.figshare.21490569.v1 [Syntax].
