# Supplementary material for: Classic psychedelics, health behavior, and physical health
Source: Ther Adv Psychopharmacol. 2022 Nov 30;12:20451253221135363. doi: 10.1177/20451253221135363 (PMC9716448; doi:10.1177/20451253221135363)
Supplement: sj-docx-1-tpp-10.1177_20451253221135363 – Supplemental material for Classic psychedelics, health behavior, and physical health [file sj-docx-1-tpp-10.1177_20451253221135363.docx]

***Supplemental Materials***

Study Description: Health behavior

The aim of this study is to better understand factors that predict health behavior. You will be required to complete a set of surveys assessing your health behaviors along with demographic measures. You will be asked sensitive questions (e.g., about substance use). To have your submission accepted, you must also correctly answer questions designed to check if you are paying attention. Anonymized data may be made available to other researchers.

**Supplemental Tables**

| Supplemental Table 1. Sample Characteristics of Lifetime Classic Psychedelic Users vs. Non-Users | | | | | |
| --- | --- | --- | --- | --- | --- |
| Variables | Users  (n = 613) | | Non-Users (n = 2209) | | *p* |
|  | % | Mean (SD) | % | Mean (SD) |  |
| **Age** |  |  |  |  | **<.001** |
| 18-25 | 13.2 |  | 17.2 |  |  |
| 26-34 | 16.0 |  | 20.3 |  |  |
| 35-49 | 22.2 |  | 25.9 |  |  |
| 50-64 | 33.1 |  | 26.1 |  |  |
| 65+ | 15.5 |  | 10.6 |  |  |
| **Gender** |  |  |  |  | **.005** |
| Male | 54.2 |  | 46.8 |  |  |
| Female | 44.2 |  | 51.6 |  |  |
| Transgender/Non-binary | 1.6 |  | 1.7 |  |  |
| **Ethnoracial identity** |  |  |  |  | **<.001** |
| Non-Hispanic White | 85.3 |  | 71.4 |  |  |
| Non-Hispanic African American | 4.4 |  | 14.5 |  |  |
| Non-Hispanic Native American/Alaska Native | 1.0 |  | 0.9 |  |  |
| Non-Hispanic Native Hawaiian/Pacific Islander | 0.0 |  | 0.2 |  |  |
| Non-Hispanic Asian | 2.8 |  | 7.3 |  |  |
| Non-Hispanic more than one race | 1.6 |  | 1.1 |  |  |
| Hispanic | 4.9 |  | 4.6 |  |  |
| **Sexual Orientation** |  |  |  |  | **.009** |
| Heterosexual | 80.9 |  | 86.2 |  |  |
| Bisexual | 10.9 |  | 7.8 |  |  |
| Gay or lesbian | 5.7 |  | 3.8 |  |  |
| Other | 2.5 |  | 2.3 |  |  |
| **Marital Status** |  |  |  |  | **.004** |
| Married / living with a partner / in a long-term relationship | 54.3 |  | 59.7 |  |  |
| Widowed | 2.9 |  | 2.6 |  |  |
| Divorced / separated | 14.7 |  | 9.8 |  |  |
| Not married / single | 28.1 |  | 27.9 |  |  |
| **Education** |  |  |  |  | **<.001** |
| Some high school or less | 0.8 |  | 0.9 |  |  |
| High school graduate or equivalent (e.g., GED) | 12.6 |  | 11.9 |  |  |
| Some college/community college degree (Associates) | 37.5 |  | 24.2 |  |  |
| Bachelor’s degree or higher | 49.1 |  | 63.0 |  |  |
| **Income** |  |  |  |  | **.007** |
| Less than US$20,000 | 12.7 |  | 11.4 |  |  |
| US$20,000–49,999 | 31.3 |  | 27.4 |  |  |
| US$50,000–74,999 | 23.0 |  | 20.5 |  |  |
| US$75,000 or more | 33.0 |  | 40.7 |  |  |
| **Engagement in risky behavior** |  |  |  |  | **<.001** |
| Never | 7.7 |  | 13.3 |  |  |
| Seldom | 39.2 |  | 46.3 |  |  |
| Sometimes | 49.8 |  | 38.5 |  |  |
| Always | 3.4 |  | 2.0 |  |  |
| **Age of first alcohol use** |  |  |  |  | **<.001** |
| Less than 13 years | 13.4 |  | 6.2 |  |  |
| 13-19 years | 76.7 |  | 59.5 |  |  |
| More than 20 years | 9.0 |  | 26.5 |  |  |
| Never used alcohol | 1.0 |  | 7.7 |  |  |
| **Lifetime tobacco use** |  |  |  |  |  |
| Lifetime smokeless tobacco use | 36.9 |  | 16.3 |  | **<.001** |
| Lifetime pipe tobacco use | 46.5 |  | 17.4 |  | **<.001** |
| Lifetime cigar use | 73.6 |  | 38.6 |  | **<.001** |
| Lifetime daily cigarette use | 63.8 |  | 26.9 |  | **<.001** |
| **Lifetime substance use** |  |  |  |  |  |
| Lifetime cocaine use | 69.3 |  | 10.2 |  | **<.001** |
| Lifetime sedative use | 52.7 |  | 23.5 |  | **<.001** |
| Lifetime pain reliever use | 83.2 |  | 61.4 |  | **<.001** |
| Lifetime marijuana use | 97.1 |  | 54.7 |  | **<.001** |
| Lifetime MDMA/ecstasy use | 41.0 |  | 4.6 |  | **<.001** |
| Lifetime PCP use | 14.2 |  | 1.4 |  | **<.001** |
| Lifetime inhalant use | 39.8 |  | 13.4 |  | **<.001** |
| **Health behavior** |  |  |  |  |  |
| Tobacco-related health behavior |  | 9.19 (1.81) |  | 9.67 (1.16) | **<.001** |
| Alcohol-related health behavior |  | 9.77 (2.62) |  | 10.72 (2.18) | **<.001** |
| High physical activity | 52.2 |  | 49.9 |  | .310 |
| Diet-related behavior |  | 8.98 (2.66) |  | 8.68 (2.64) | **.012** |
| **Markers of physical health** |  |  |  |  |  |
| Healthy BMI | 34.8 |  | 38.1 |  | .132 |
| NCD in the past year | 22.0 |  | 17.2 |  | **.006** |
| All percentages were rounded to the nearest 0.1%; cumulative percentages may not add to 100.0. SD = Standard Deviation. MDMA: 3,4-methylenedioxymethamphetamine; PCP: phencyclidine. Pearson χ2 tests and independent t-tests were used to examine the characteristics of lifetime classic psychedelic users vs. non-lifetime classic psychedelic users. | | | | | |

It is important to note that ethnoracial identity was assessed with a single-item question (“Which of these groups describes you?” Response options: 1) Non-Hispanic White, 2) Non-Hispanic African American, 3) Non-Hispanic Native American/Alaska Native, 4) Non-Hispanic Native Hawaiian/Pacific Islander, 5) Non-Hispanic Asian, 6) Non-Hispanic more than one race, 7) Hispanic), which differs slightly from census-based assessments of ethnicity. The distribution of ethnicity in this study’s sample, based on the five categories used by Prolific Academic to stratify the sample, was the following: Asian (6.4%), Black (12.9%), Mixed (2.6%), Other (2.2%), White (75.9%).

*Frequency of use and age of first use*

Respondents who reported lifetime classic psychedelic use were asked to report how old they were the first time they used a classic psychedelic and how many times they had used classic psychedelics in their lifetime (1, 2-5, 6-10, 11-20, 21-50, 51-100, 101-300, More than 300 times).

| Supplemental Table 2. Frequency of use, age of first use, and health behavior | | | | | | | | | |
| --- | --- | --- | --- | --- | --- | --- | --- | --- | --- |
|  | Tobacco | | Alcohol | | Diet | | Exercise | |  |
|  | *β* | *p* | *β* | *p* | *β* | *p* | aOR (CI 95%) | *p* | *N* |
| Frequency of use | .03 | .429 | -.02 | .631 | .11 | **.014** | 0.99 (0.89-1.11) | .902 | 613 |
|  |  |  |  |  |  |  |  |  |  |
| Age of first use | .00 | .973 | -.08 | .065 | -.04 | .410 | 1.00 (0.98-1.02) | .887 | 613 |
| β = standardized coefficients; aOR = adjusted Odds Ratios; β and aORs are adjusted for age in years, gender, ethnoracial identity, sexual orientation, educational attainment, annual household income, marital status, self-reported engagement in risky behavior, lifetime use of cocaine, sedatives, pain relievers, marijuana, phencyclidine (PCP), 3,4-methylenedioxymethamphetamine (MDMA/ecstasy), inhalants, smokeless tobacco, pipe tobacco, cigars, and cigarettes daily, and age of first alcohol use. | | | | | | | | | |

| Supplemental Table 3. Frequency of use, age of first use, and physical health | | | | | |
| --- | --- | --- | --- | --- | --- |
|  | Healthy BMI | | NCD in the past year | |  |
|  | aOR (CI 95%) | *p* | aOR (CI 95%) | *p* | *N* |
| Frequency of use | 0.96 (0.85-1.09) | .558 | 1.05 (0.91-1.21) | .513 | 613 |
|  |  |  |  |  |  |
| Age of first use | 1.03 (1.00-1.05) | **.042** | 0.97 (0.93-1.00) | .062 | 613 |
| aOR = adjusted Odds Ratios; aOR are adjusted for age in years, gender, ethnoracial identity, sexual orientation, educational attainment, annual household income, marital status, self-reported engagement in risky behavior, lifetime use of cocaine, sedatives, pain relievers, marijuana, phencyclidine (PCP), 3,4-methylenedioxymethamphetamine (MDMA/ecstasy), inhalants, smokeless tobacco, pipe tobacco, cigars, and cigarettes daily, and age of first alcohol use. Note: due to empty cells and collinearity in Stata, educational attainment was dropped from regression with frequency of use and age of first use and NCD in the past year. | | | | | |
